# MemLoTrack: Enhancing TIR Anti-UAV Tracking with Memory-Integrated Low-Rank Adaptation

**DOI:** 10.3390/s25237359

**Published:** 2025-12-03

**Authors:** Jae Kwan Park, Ji-Hyeong Han

**Affiliations:** 1 Department of Defense AI Convergence Engineering, Seoul National University of Science and Technology, Seoul 01811, Republic of Korea; 16102241@seoultech.ac.kr; 2 Department of Computer Science and Engineering, Seoul National University of Science and Technology, Seoul 01811, Republic of Korea

**Keywords:** anti-UAV, thermal infrared (TIR), single-object tracking (SOT), low-rank adaptation (LoRA), memory attention layer (MAL), gated memory bank (MB), vision transformer (ViT), DINOv2

## Abstract

Tracking small, fast-moving unmanned aerial vehicles (UAVs) in thermal infrared (TIR) imagery is a significant challenge due to low-resolution targets, Dynamic Background Clutter, and frequent occlusions. To address this, we introduce MemLoTrack, a novel onestream Vision Transformer tracker that integrates a memory mechanism into a parameterefficient LoRA framework. MemLoTrack enhances a baseline tracker (LoRAT) with two key components: (i) a gated First-In, First-Out (FIFO) memory bank (MB) for temporal context aggregation and (ii) a lightweight Memory Attention Layer (MAL) for effective information retrieval. A key component of our method is a selective memory update policy, which commits a frame to the memory bank only when it satisfies both a classification confidence threshold (τ) and a Kalman filter-based motion consistency check. This gating mechanism robustly prevents memory contamination due to distractors, occlusions, and reappearance events. Our training is highly efficient, updating only the LoRA adapters, MAL, and prediction head while the pretrained DINOv2 backbone remains frozen. Evaluated on the challenging Anti-UAV410 benchmark, MemLoTrack (*L*_mem_ = 7, τ = 0.8) achieves an AUC of 63.6 and a State Accuracy (SA) of 64.0, representing a significant improvement over the LoRAT baseline by +1.4 AUC and +1.5 SA. Compared to the state-of-the-art method FocusTrack, MemLoTrack demonstrates superior robustness with higher AUC (63.6 vs. 62.8) and SA (64.0 vs. 63.9), while trading lower precision (P/P-Norm) scores. Furthermore, MemLoTrack operates at 153 FPS on a single RTX 4070 Ti SUPER, demonstrating that parameter-efficient fine-tuning with a selective memory mechanism is a powerful and deployable strategy for real-time Anti-UAV tracking in demanding TIR environments.

## 1. Introduction

Thermal infrared (TIR) single-object tracking (SOT) plays a crucial role in Anti-UAV systems, ensuring continuous perimeter surveillance and airspace security, especially under low illumination and adverse weather conditions [1,2]. Despite remarkable advances in recent years, TIR tracking remains highly challenging due to the low resolution and texture deficiency of aerial targets, frequent Thermal Crossover events, and abrupt viewpoint variations arising from platform motion [2,3]. In such complex conditions, local search-based trackers often fail when the target undergoes occlusion or abrupt motion, whereas global re-detection strategies, though more resilient, incur a substantial computational cost [4,5,6]. The Anti-UAV410 benchmark explicitly exposes these long-term robustness limitations through its challenging TIR sequences and attributes, including Thermal Crossover (TC), Dynamic Background Clutter (DBC), and occlusion (OC) [2].

Transformer-based one-stream trackers, which perform joint encoding of the template (*z*) and search region (*x*), have emerged as a dominant SOT paradigm by enabling early feature interaction [6,7,8,9]. Concurrently, parameter-efficient fine-tuning (PEFT) methods, notably Low-Rank Adaptation (LoRA), allow large Vision Transformer (ViT) backbones to be adapted with minimal computational cost by freezing most weights [10,11]. This efficiency is highly desirable for resource-constrained Anti-UAV systems. For instance, LoRAT [12] applies LoRA modules to a ViT backbone, demonstrating a path toward balancing tracking accuracy with computational throughput.

However, existing local trackers, including LoRAT, exhibit two major failure modes when evaluated on the challenging Anti-UAV410 dataset. First, abrupt camera motion can easily shift the small UAV target completely outside the predefined search region. Second, and more critically, the absence of a robust temporal memory mechanism causes identity drift during occlusion, out-of-view situations, and severe background clutter [3,13]. Although several approaches attempt to enhance robustness through adaptive or focused sampling, the fundamental issue of preserving long-term temporal coherence remains largely unresolved.

To overcome these limitations, we propose MemLoTrack, a LoRA-based, one-stream ViT tracker that incorporates an explicit, dual-gated memory bank (MB). Our approach freezes a high-capacity DINOv2 ViT encoder and trains only the low-rank adapters, a lightweight Memory Attention Layer (MAL) and an MLP-based prediction head [14]. The core of our innovation lies in the memory update policy: the feature tokens of a given frame are written to the MB only if two conditions are met: (i) the classification confidence exceeds a threshold τ, and (ii) a Kalman filter-based motion consistency test is passed. This dual-gating mechanism effectively prevents memory pollution from distractors and ambiguous observations. At inference, the MAL cross-attends the current search region tokens to the curated memory tokens, thereby mitigating drift with minimal computational overhead. Our design is informed by prior work showing that gated, attention-based memory policies significantly outperform naive fixed-window buffers in challenging tracking scenarios [13].

The main contributions of this work are summarized as follows:

- **Memory-Integrated PEFT Tracker:** We introduce a parameter-efficient ViT architecture featuring a Memory Attention Layer (MAL) that leverages long-range temporal context while preserving the efficiency of LoRA-based fine-tuning.
- **Dual-Gated Memory Write Policy:** During inference, a compact FIFO memory bank is governed by a novel dual-gated policy that admits frames only upon passing both a confidence check and a Kalman-based motion consistency check. This strategy robustly mitigates memory contamination under occlusion (OC), out-of-view (OV), and Dynamic Background Clutter (DBC) situations.
- **State-of-the-Art Robustness on Anti-UAV410:** MemLoTrack achieves state-of-the-art (SOTA) performance on the Anti-UAV410 benchmark, attaining an AUC of 63.6 and an SA of 64.0. These metrics, which reflect overall success and re-acquisition capabilities, establish a new benchmark for tracking robustness. While FocusTrack [3] demonstrates superior precision-based metrics (P/P-Norm), our method presents a notable advancement in robustness. This advancement is primarily attributed to our dual-gated memory policy, which effectively curates the temporal context, prevents memory contamination, and significantly enhances target re-acquisition during critical failure modes such as occlusion (OC), out-of-view (OV), and Dynamic Background Clutter (DBC).

The remainder of this paper is organized as follows. Section 2 reviews related work. Section 3 details the proposed architecture. Section 4 presents the experimental setup, ablation studies, and results.

## 2. Related Work

We review three categories of prior work most pertinent to MemLoTrack: (i) transformer based single-object tracking (SOT) with parameter-efficient adaptation; (ii) Anti-UAV tracking in thermal infrared (TIR), including benchmarks and protocol details; and (iii) memory-augmented tracking and motion-aware memory selection that motivate our memory bank (MB) and Memory Attention Layer (MAL) design.

### 2.1. Transformer-Based SOT and Parameter-Efficient Adaptation

**One-stream ViT trackers and LoRA-based fine-tuning.** Transformer backbones have become standard in modern SOT because self-attention enables long-range dependency modeling and early fusion of template–search tokens. One-stream trackers concatenate template and search before multi-head self-attention, improving interaction compared with two-stream variants [3,6,8,15,16]. LoRAT [12] operationalizes low-rank adaptation (LoRA) for this one-stream paradigm: it freezes the ViT backbone [14], inserts LoRA into attention blocks, introduces token-type embeddings to disambiguate template vs. search tokens, and replaces the convolutional head with a lightweight MLP head to avoid inductive-bias conflicts under PEFT [10,12,17,18,19]. LoRAT demonstrates competitive accuracy–efficiency trade-offs on several SOT benchmarks, including LaSOT [20], LaSOT-Ext/TrackingNet [21], GOT-10k [22], and TNL2k [23]. Notably, LoRAT-B-224 achieves high throughput, with certain variants reaching up to 603 fps on a NVIDIA RTX 4090, illustrating that adapter-based one-stream tracking is both practical and accurate [10,12].

### 2.2. Anti-UAV Tracking in TIR: Benchmarks, Protocols, and Specialized Designs

**Benchmarks and evaluation protocol.** Anti-UAV300 (RGB–T) [1] and Anti-UAV410 (TIR-only) [2] are the benchmarks for vision-based Anti-UAV tracking. Anti-UAV [1,2] defines the State Accuracy (SA) metric that integrates IoU with target-visibility flags (predict “present/absent”) and also reports Success AUC and precision; Anti-UAV410 follows the same core protocol—Success AUC and precision and adopts SA for comparability. Normalized precision (P-Norm) is frequently reported by subsequent works for completeness but is not used as the official ranking metric in the Anti-UAV410 paper. Both benchmarks provide attribute-wise evaluation—Thermal Crossover (TC), Dynamic Background Clutter (DBC), Fast Motion (FM), occlusion (OC), Out-of-View (OV), and Scale Variation (SV)—and size categories, enabling fine-grained analysis of failure modes [1,2].

**RGB vs. TIR characteristics in Anti-UAV tracking.** While RGB trackers capitalize on detailed visual features available in the visible spectrum, they remain inherently vulnerable to environmental factors such as sudden illumination shifts and adverse weather. TIR tracking overcomes these photometric limitations but introduces unique challenges: the target UAV is often an indistinct, textureless blob, frequently merging with background thermal patterns. This inherent ambiguity, exacerbated by sensor noise and Thermal Crossover, suggests that the tracking paradigm should deviate from appearance-heavy matching. As a result, successful Anti-UAV trackers must combine per-frame appearance cues with temporally aggregated evidence and motion priors to resolve target identity under dynamic clutter and long-range localization difficulties, regardless of modality. In this work, we therefore adopt a unified one-stream ViT backbone augmented with an explicit memory bank and a Kalman-based motion prior, and we empirically show in Section 4 that this memory-integrated temporal design yields clear robustness gains on the TIR-only Anti-UAV410 and Anti-UAV300 IR benchmarks, while offering only limited benefits on RGB Anti-UAV data, reflecting its specialization for thermal infrared Anti-UAV tracking.

**Vision-based Anti-UAV benchmarks and pipelines.** Beyond the scope of Anti-UAV410, vision-based Anti-UAV tracking has been extensively explored on RGB and RGB-T benchmarks [1,24]. These studies typically employ detection and tracking cascade pipelines that couple high-performance UAV detectors with multi-object trackers. A key advantage of this framework is the ability to trigger global re-detection whenever tracker confidence drops, thereby recovering lost targets. In contrast to this detection-centric approach, a parallel line of research focuses on lightweight SOT models based on Transformer backbones that operate within restricted search regions instead of performing full-frame scanning. MemLoTrack aligns with this latter paradigm. It integrates temporal memory and a Kalman prior into a single-object Transformer tracker to maintain trajectories without relying on external detectors. Consequently, our experimental design applies this identical framework to both a standard RGB Anti-UAV benchmark and the TIR-only Anti-UAV410 dataset. While the former provides comparative validation against competitive local trackers, the latter is dedicated to rigorous attribute-wise analyses and ablations to stress-test robustness against tiny, low-contrast targets and challenging occlusions.

**Task characteristics and domain-tailored trackers.** Anti-UAV410 emphasizes long-range imaging of tiny UAVs in natural scenes (forests, mountains, lakes), increasing background clutter and target ambiguity; the dataset explicitly highlights DBC and tiny-size prevalence, making local tracker design challenging [2]. Within local tracker lines, a recent Anti-UAV method (FocusTrack) demonstrates that self-adaptive search-region adjustment (SRA) and hierarchical attention-to-mask aggregation can substantially narrow the gap to global re-detection while preserving throughput—evidence that local tracking can be competitive on Anti-UAV410 if its field of view is adaptively controlled [3]. On the TIR-specialized side, FFTR augments a ViT-style encoder with frequency-channel attention and a template-reconstruction mechanism; by training compact adapter-like modules rather than fully fine-tuning the backbone, FFTR shows strong results on PTB-TIR [25], LSOTB-TIR [26], and Anti-UAV410, underscoring the value of domain priors and lightweight temporal updating for IR data [27].

### 2.3. Memory-Augmented Tracking and Motion-Aware Memory Selection

**Streaming memory for video perception.** External key–value memory is a long-standing mechanism to maintain identity through occlusions and appearance changes. SAM 2 adopts a streaming memory architecture that stores past frame embeddings and performs memory attention for promptable video segmentation; ablations indicate that direct memory embeddings with appropriate positional encoding strike a favorable speed–accuracy balance [28]. These insights motivate our MB–MAL design while we target SOT rather than segmentation.

**Motion-aware Single-Object Tracking (SOT).** Recent SOT frameworks have also started to model target motion explicitly at the sequence level. SwinTrack [9] introduces a dedicated motion token that summarizes past trajectories and injects it into the Transformer backbone, improving robustness under Fast Motion. ODTrack [29] learns dense temporal tokens over consecutive search regions so that motion patterns are embedded into the token sequence and updated online. ARTrack [16] treats tracking as an autoregressive trajectory prediction problem, tokenizing past bounding boxes and feeding them as spatio-temporal prompts to a Transformer decoder. These methods demonstrate that mixing appearance features with motion-aware tokens is an effective way to stabilize long-term trajectories. Our MemLoTrack is complementary to this line: instead of replacing the localization head with an autoregressive decoder, we augment a strong one-stream PEFT-based tracker with a gated memory bank and a Kalman prior tailored to TIR Anti-UAV410 [2].

**Motion-aware memory selection.** Selective write policies are crucial: naive FIFO buffers (recent-*N* windows) admit low-quality frames under occlusion, Fast Motion, or viewpoint changes, which degrades long-term memory. Building on SAM 2’s streaming memory, SAMURAI replaces this fixed-window FIFO with a hybrid scoring strategy that combines mask affinity, objectness, and kalman-based motion cues when deciding which frames to store—yielding higher success and precision without task-specific fine-tuning [13]. Motivated by these findings, we apply dual gating—(i) a classification confidence filter and (ii) a Kalman-based motion consistency check—before writing to the memory bank (MB), curbing error propagation from irregular updates and motion-induced drift.

**Positioning of MemLoTrack.** Within this landscape, MemLoTrack starts from a one-stream ViT tracker with LoRA confined to self-attention (PEFT) and adds an explicit MAL between the encoder and head. During training, the model samples seven memory frames between the template and the current search region frame to align optimization with the memory-augmented inference pipeline; at inference, a FIFO MB stores post-embedding tokens and supplies K, V to MAL, but only after dual gating. Our experiments on Anti-UAV410 follow the official success/precision/SA protocol (we additionally report P-Norm) [1,2].

## 3. Method

### 3.1. Backbone and Trainable Scope

**Figure 1:**
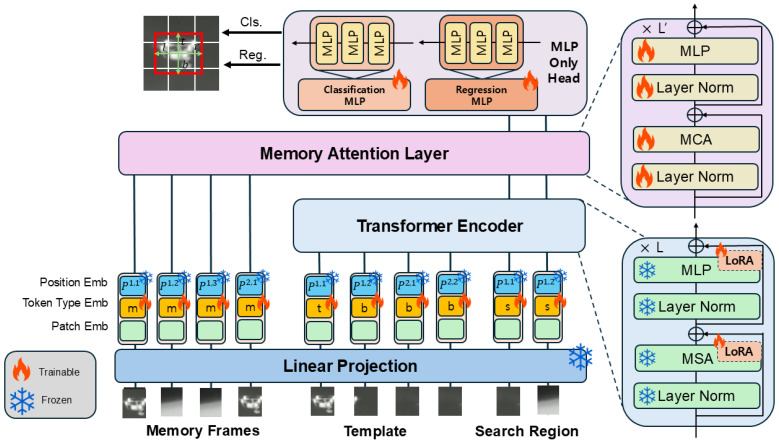
Training pipeline. The template *z* and the current search $x_{t}$ are jointly encoded (frozen DINOv2 + LoRA). We sample seven memory frames from the interval between *z* and $x_{t}$ and feed their post-embedding tokens to MAL as keys/values. No runtime memory bank is maintained during training.

MemLoTrack is a one-stream ViT tracker that jointly encodes the template *z* and the search frame $x_{t}$. The backbone is a frozen DINOv2 ViT backbone (patch size p=14, embedding width d=768). Parameter-efficient adaptation is achieved by inserting LoRA adapters (rank r=64) into all linear projections of attention and MLP blocks. The set of trainable components consists of (1) the LoRA adapters, (2) a Memory Attention Layer (MAL) positioned between the encoder and the MLP head, (3) learned token-type embeddings (template foreground/background/search region/memory frames), and (4) a lightweight MLP-only prediction head. During training, we jointly encode $\left(z,x_{t}\right)$ to obtain the search queries $X_{t}$. We then sample seven memory frames that lie between *z* and $x_{t}$. Each sampled frame is converted to tokens using the same patch embedding as the search, an absolute positional table on the token grid, and the memory type embedding. These memory frame tokens bypass the encoder and are stacked as keys/values for MAL, while $X_{t}$ serves as queries. Neither a memory bank nor gates are used during training. The head operates on the token grid of the search region and produces a classification map and four distances per cell. Figure 1 summarizes this process.

### 3.2. Inputs and Tokens

Each training instance consists of a template $z\;\in \;R^{3\times 112\times 112}$, a search frame $x_{t}\;\in \;R^{3\times 224\times 224}$, and $L_{\mathrm{mem}}$ memory frames $\left\{m_{i}\right\}$ at the search resolution. Let PE(·) denote the frozen ViT patch embedding and $P^{\left(a,b\right)}\;\in \;R^{(ab)\times d}$ an absolute 2D positional table shared across sources. We use four learnable token-type embeddings: $e_{t},e_{b},e_{s},e_{m}\;\in \;R^{d}$.

**Input Token Formulation.** The final input tokens are constructed by combining the patch embedding PE(·), the shared positional embedding $P^{\left(i,j\right)}$ for grid position (i,j), and the appropriate token-type embedding. Let $T^{\left(i,j\right)}$, $S^{\left(i,j\right)}$, and $M^{\left(i,j\right)}$ be the image patches at grid position (i,j) for the template, search, and memory inputs, respectively. The resulting tokens are formally defined as follows:

(1) $$\begin{array}{cc} E_{T}^{\left(i,j\right)} & =\mathrm{PE}\left(T^{\left(i,j\right)}\right)+P^{\left(i,j\right)}+\left\{\begin{array}{cc} \;e_{t}, & \mathrm{if}\;\mathrm{patch}\;(i,j)\;\mathrm{is}\;\mathrm{target}\;\left(t\right)\\ \;e_{b}, & \mathrm{otherwise}\;\left(b\right) \end{array}\right. \end{array}$$

(2) $$\begin{array}{cc} E_{S}^{\left(i,j\right)} & =\mathrm{PE}\left(S^{\left(i,j\right)}\right)+P^{\left(i,j\right)}+e_{s} \end{array}$$

(3) $$\begin{array}{cc} E_{M}^{\left(i,j\right)} & =\mathrm{PE}\left(M^{\left(i,j\right)}\right)+P^{\left(i,j\right)}+e_{m} \end{array}$$

These individual tokens are then flattened to form the template sequence $T_{z}\in R^{B\times N_{z}\times d}$ and the search sequence $T_{x_{t}}\in R^{B\times N_{x}\times d}$, where $N_{z}=\left(H_{z}/p\right)\left(W_{z}/p\right)$ and $N_{x}=\left(H_{x}/p\right)\left(W_{x}/p\right)$. The memory tokens (from Equation (3)) are processed separately and do not enter the main encoder.

**Encoder output.** Concatenating $T_{z}$ and $T_{x_{t}}$ and passing them through the encoder yields the search queries $X_{t}\in R^{B\times N_{x}\times d}$ that are later conditioned by MAL.

**Notation for memory tokens.** To avoid confusion with the confidence threshold τ, we use the following convention throughout the paper: (i) the current frame’s MB write candidate is $E_{t}\in R^{B\times N_{x}\times d}$; (ii) any past entry already stored in the MB is written as $E_{t^{\prime }}$. The stacked memory at time *t* is $M_{t}=\mathrm{concat}\left(E_{t_{1}^{\prime }},\ldots ,E_{t_{n_{t}}^{\prime }}\right)\in R^{B\times N_{m}\times d}$ with $N_{m}=n_{t}\cdot N_{x}$ and $n_{t}\leq L_{\mathrm{mem}}$.

### 3.3. Training-Time Memory Frame Sampling

**Figure 2:**
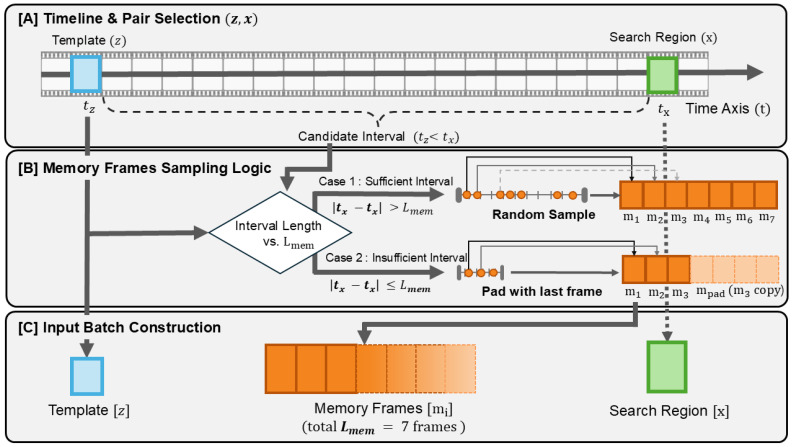
Memory frame sampling strategy during training. The pipeline illustrates (**A**) the selection of a template (*z*) and a search region (*x*) on the timeline, (**B**) the memory frame sampling logic that applies random sampling for sufficient intervals or pads with the last frame for insufficient ones, and (**C**) the construction of the final input batch with a fixed memory length $L_{mem}$.

To align the training process with our inference-time memory mechanism, we adopt a streaming-based memory sampling strategy inspired by recent work in large-scale video understanding, such as SAM 2 [28]. For each training instance, a template frame *z* at time $t_{z}$ and a current search frame *x* at $t_{x}$ are first sampled. Subsequently, we determine the set of memory frames based on the temporal interval between $t_{z}$ and $t_{x}$, as illustrated in Figure 2. Specifically, the sampling logic branches into two cases depending on the interval length relative to the memory budget $L_{\mathrm{mem}}$: (1) If the interval is sufficient ($|t_{x}-t_{z}|>L_{\mathrm{mem}}$), we randomly sample $L_{\mathrm{mem}}$ distinct frames from the intermediate period to cover diverse temporal contexts. (2) If the interval is insufficient ($|t_{x}-t_{z}|\leq L_{\mathrm{mem}}$), we utilize all available intermediate frames and pad the remaining slots by duplicating the last valid frame ($m_{\mathrm{pad}}$). Each selected frame is tokenized and directly supplied as keys and values to the MAL.

During training, these seven frames act as an ephemeral memory. They are used only within the current mini-batch and are not stored in a persistent buffer over time or across batches. We fix the number of sampled memory frames to seven so that the MAL is always exposed to the same context length as the default inference-time budget $L_{\mathrm{mem}}=7$. In contrast, during inference, MemLoTrack maintains a FIFO memory bank of size $L_{\mathrm{mem}}$ that accumulates post-embedding tokens $E_{t}$ over time whenever the dual gates are satisfied. Rather than explicitly unrolling this recurrent bank during training, we follow the streaming formulation of SAM 2 [28] and train the MAL on fixed-size snippets of intermediate frames that share the same embedding format as the runtime memory tokens.

### 3.4. Memory Attention Layer (MAL)

MAL performs cross-attention from the search queries $X_{t}$ to the stacked memory tokens $M_{t}$ and outputs a memory-conditioned representation:

(4) $$\mathrm{Attn}\left(X,M\right)=\mathrm{softmax}\;\left(\frac{\left(XW^{Q}\right){\left(MW^{K}\right)}^{⊤}}{\sqrt{d_{h}}}\right)\;\left(MW^{V}\right)\;W^{O}.$$

If the memory stack is empty, such as at the beginning of a sequence, the MAL is bypassed and the query tokens $X_{t}$ are sent directly to the MLP head.

### 3.5. Inference-Time Memory Bank (MB) with Dual Gating

At inference, we maintain a FIFO memory bank of capacity $L_{\mathrm{mem}}$. After processing frame *t*, two gates are applied to decide whether to write the current entry: (i) a confidence gate that accepts if the maximum classification score $s_{t}$ exceeds a threshold τ; and (ii) a motion consistency gate based on a Kalman prediction that accepts if the Mahalanobis distance between the predicted box and the decoded box is below a chi-square threshold. If a frame is accepted, its tokens $E_{t}$ are first spatially aligned to the current query grid via an affine transformation and then added to the bank. This alignment ensures that memory tokens and current queries share a canonical coordinate system.

(5) $$E_{t}={\mathrm{Align}}_{t}\left(\mathrm{PE}\left(x_{t}\right)\right)+P_{16\times 16}+e_{\mathrm{mem}}\in R^{B\times N_{x}\times d}.$$

If the memory bank is full, the oldest entry is evicted. When the bank is non-empty, its tokens $\left(M_{t}\right)$ serve as MAL keys/values for the next frame; if it is empty, MAL is bypassed. Alignment uses the crop-to-crop affine transform (axis-aligned scaling and translation) between that frame’s search window and the current one; if unavailable, we fall back to an identity warp. This ensures that memory tokens and current queries share the same canonical 16×16 grid.

**Dual gating policy.** Let $p_{t}\in {\left[0,1\right]}^{N_{x}}$ denote the classification scores over the $N_{x}=16\times 16$ spatial locations at frame *t*, and define

$$s_{t}=\max_{1\leq i\leq N_{x}}p_{t}\left(i\right).$$

The confidence gate accepts frame *t* if

$$s_{t}\geq \tau ,$$

where τ∈(0,1) is an inference-time threshold selected on the Anti-UAV410 validation split.

For motion consistency, we maintain a constant-velocity Kalman filter per target with 6-D state vector:

$$x_{t}={\left[c_{x},c_{y},v_{x},v_{y},\log w,\log h\right]}^{⊤}\in R^{6},$$

where $\left(c_{x},c_{y}\right)$ and (w,h) are the box center and size, and measurement vector

$$z_{t}={\left[c_{x},c_{y},\log w,\log h\right]}^{⊤}\in R^{4}$$

is obtained from the decoded box at frame *t*. Given the predicted state $\left({\hat{x}}_{t},{\hat{P}}_{t}\right)$ before seeing $z_{t}$, the innovation and its covariance are

$$y_{t}=z_{t}-H{\hat{x}}_{t},\;\Sigma_{t}=H{\hat{P}}_{t}H^{⊤}+R,$$

where $H\in R^{4\times 6}$ selects the $\left(c_{x},c_{y},\log w,\log h\right)$ components of the state, and $R\in R^{4\times 4}$ is the measurement covariance. The Mahalanobis distance between prediction and measurement is then

$$d_{t}^{2}=y_{t}^{⊤}\Sigma_{t}^{-1}y_{t}.$$

The Kalman motion gate accepts the measurement if

$$d_{t}^{2}<\lambda ,$$

where λ>0 is a chi-square threshold (initially set to the 95%th percentile for four degrees of freedom). In addition, we discard clearly implausible measurements whose IoU with the predicted box is too small, whose scale ratio is outside a reasonable range, or whose center displacement exceeds a predefined velocity bound; these simple geometric checks are applied before evaluating $d_{t}^{2}$.

A frame is finally written to the memory bank only if it passes *both* gates, i.e., $s_{t}\geq \tau$ and $d_{t}^{2}<\lambda$, together with the geometric consistency checks above. This dual gating policy prevents low-confidence or kinematically inconsistent frames from polluting the memory while keeping the mechanism lightweight for real-time deployment.

### 3.6. Loss and Optimization

The trainable components of our model—specifically, the LoRA adapters, the Memory Attention Layer (MAL), and the prediction head—are optimized jointly in an end-to-end manner. The total loss function L is formulated as a weighted sum of a binary cross-entropy (BCE) term for classification and a Generalized Intersection over Union (GIoU) term for bounding box regression:

(6) $$L=\lambda_{\mathrm{cls}}\cdot \mathrm{BCE}\;\left(S_{t},S_{t}^{★}\right)\;+\;\lambda_{\mathrm{box}}\cdot \left(1-\mathrm{GIoU}({\hat{B}}_{t},B_{t}^{★})\right).$$

For both training and inference, the template and search region sizes are configured to z=112×112 and x=224×224, respectively. The MB capacity is fixed at $L_{\mathrm{mem}}=7$ during inference to maintain consistency with the seven memory frames sampled per instance during training.

### 3.7. Prediction Head and Targets

The head is a lightweight MLP applied per token on the 16×16 token grid produced by the encoder (each token corresponds to a 14×14 pixel region in the 224×224 search window). For every grid cell, it outputs a classification score $S_{t}$ and four distances (ℓ,t,r,b). Box formation converts these distances into a rectangle around the cell center $\left(c_{x},c_{y}\right)$:

(7) $$x_{L}=c_{x}-\ell ,\;x_{R}=c_{x}+r,\;y_{T}=c_{y}-t,\;y_{B}=c_{y}+b.$$

Targets $\left(S_{t}^{★},B_{t}^{★}\right)$ are assigned on a center-based (anchor-free) grid.

### 3.8. Inference Procedure

**Figure 3:**
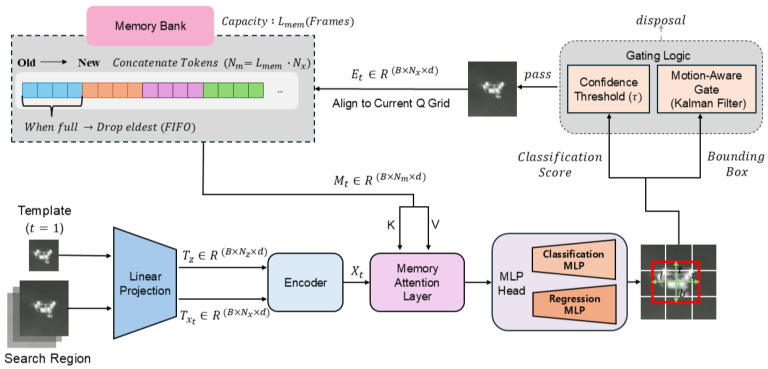
Inference pipeline with runtime memory bank. After head decoding, two gates are evaluated. Only if both pass, the frame is aligned to the current query grid and its post-embedding tokens $E_{t}$ are written into the FIFO memory bank (capacity $L_{\mathrm{mem}}$; drop oldest when full). On subsequent frames, bank tokens serve as K,V for MAL; if the bank is empty, MAL is bypassed.

The procedure follows Figure 3. (i) Form the search queries $X_{t}$ by jointly encoding $\left(z,x_{t}\right)$. (ii) If the memory bank is non-empty, apply MAL with bank tokens as K,V to obtain ${\tilde{X}}_{t}$; otherwise bypass MAL and set ${\tilde{X}}_{t}=X_{t}$. (iii) Decode the classification map and the bounding box. (iv) Apply the confidence gate using the maximum score. (v) Apply the motion consistency gate using the Kalman-based Mahalanobis test. (vi) If either gate fails, discard the frame (no write). If both pass, align to the current query grid and write the tokens $E_{t}$ to the FIFO bank. (vii) Proceed to the next frame, where the bank supplies K,V to MAL.

## 4. Experiments and Results

### 4.1. Anti-UAV410 Protocol and Metrics

We follow the Anti-UAV410 [2] one-pass evaluation (OPE) protocol on thermal infrared sequences, which include attribute annotations for Thermal Crossover (TC), Dynamic Background Clutter (DBC), Fast Motion (FM), occlusion (OC), Out-of-View (OV), and Scale Variation (SV), as well as target-size buckets (Tiny, Small, Medium, and Normal). Performance is quantified using four standard metrics. First, Success AUC assesses overall robustness, defined as the area under the success rate–IoU curve, where the success rate represents the proportion of frames satisfying a given Intersection-over-Union (IoU) threshold. Second, precision (P) measures localization accuracy based on the 20-pixel criterion, specifically calculating the percentage of frames where the center location error relative to the ground truth is less than 20 pixels. Third, to mitigate scale sensitivity, we report Normalized Precision ($P_{\mathrm{Norm}}$), which normalizes the center location error by the ground truth bounding box size prior to thresholding. Finally, we employ State Accuracy (SA) to address the target visibility fluctuations inherent in UAV tracking. Unlike conventional metrics, SA explicitly incorporates the “target present/absent” status. It evaluates IoU-based localization for visible targets while assessing the tracker’s presence prediction accuracy for absent ones. This is formulated as follows:

(8) $$SA=\frac{1}{T}\sum_{t=1}^{T}\left({\mathrm{IoU}}_{t}\cdot \delta \left(v_{t}>0\right)+p_{t}\cdot \left(1-\delta \left(v_{t}>0\right)\right)\right),$$

where *T* denotes the sequence length, ${\mathrm{IoU}}_{t}$ is the IoU with the ground truth, $v_{t}$ represents the ground truth visibility flag, and $p_{t}$ is the predicted presence probability. We adopt two evaluation regimes:non-training (using pre-trained weights) and re-training (fine-tuned on Anti-UAV410). Unless stated otherwise, input resolutions are z=112×112 and x=224×224. For inference, the memory bank capacity is fixed at seven frames to match the training configuration (see Section 3.3), with a memory update confidence threshold of τ=0.8 determined via validation.

### 4.2. Anti-UAV300 Training and Evaluation

We extended our evaluation to the Anti-UAV300 IR benchmark [1]. All evaluated trackers were fine-tuned on the Anti-UAV300 IR train split following the protocol described in Section 4.1. As shown in Table 1, MemLoTrack consistently outperforms the strong LoRAT-B [12] (66.4 AUC, 64.8 SA). Specifically, the configuration with MB=11 improves performance to 66.9 AUC and 66.6 SA. This enhancement stems from the memory-integrated adaptation pipeline, where PEFT-based fine-tuning and confidence-motion gating jointly adapt the model to dataset-specific temporal patterns. Notably, the gains are more pronounced in State Accuracy (SA) than in AUC. This indicates that the proposed method primarily bolsters temporal consistency and visibility handling while retaining the robust single-frame localization capabilities inherited from LoRAT-B [12].

**Table 1:** Performance comparison of trackers on the Anti-UAV300 IR test set. The metrics include Area Under Curve (AUC), precision (P), and State Accuracy (SA).

Method	AUC	P	SA
ATOM [30]	49.2	68.0	50.0
TransT [31]	51.3	68.1	52.1
Super DiMP [32]	55.8	77.2	56.7
STARK [33]	58.2	76.8	59.1
GlobalTrack [4]	63.3	85.5	64.3
LoRAT-B [12]	66.4	85.6	64.8
**MemLoTrack (MB = 7)**	66.6	86.3	66.4
**MemLoTrack (MB = 11)**	66.9	86.7	66.6

### 4.3. Overall Comparison on Anti-UAV410 and Cross Dataset Evaluation

Table 2 reports the Anti-UAV410 test set comparison of MemLoTrack against recent state-of-the-art trackers under both non-training (pre-trained) and re-training (fine-tuned) regimes. Under the re-training setting, MemLoTrack (MB =7, τ=0.8, x=224×224) achieves AUC 63.6, P 82.7, P-Norm 79.8, and SA 64.0. Relative to FocusTrack, MemLoTrack improves AUC (62.8 → 63.6) and SA (63.9 → 64.0) while achieving lower scores on P (86.2 vs. 82.7) and P-Norm (82.8 vs. 79.8). Compared with the LoRAT-B baseline at the same resolution (AUC 62.2, P 80.9, P-Norm 78.1, SA 62.5), MemLoTrack yields consistent gains across all four metrics (+1.4 AUC, +1.8 P, +1.7 P-Norm, +1.5 SA).

**Figure 4:**
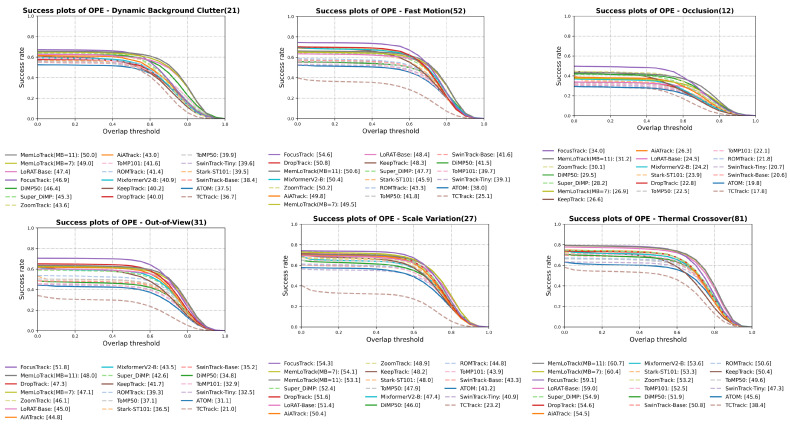
Attribute-wise Success AUC on Anti-UAV410 (DBC, TC, SV, OC, OV, FM).

**Figure 5:**
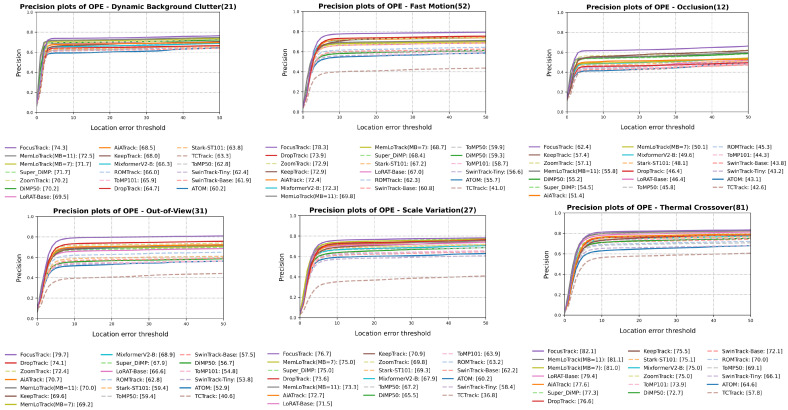
Attribute-wise precision on Anti-UAV410 (DBC, TC, SV, OC, OV, FM).

**Figure 6:**
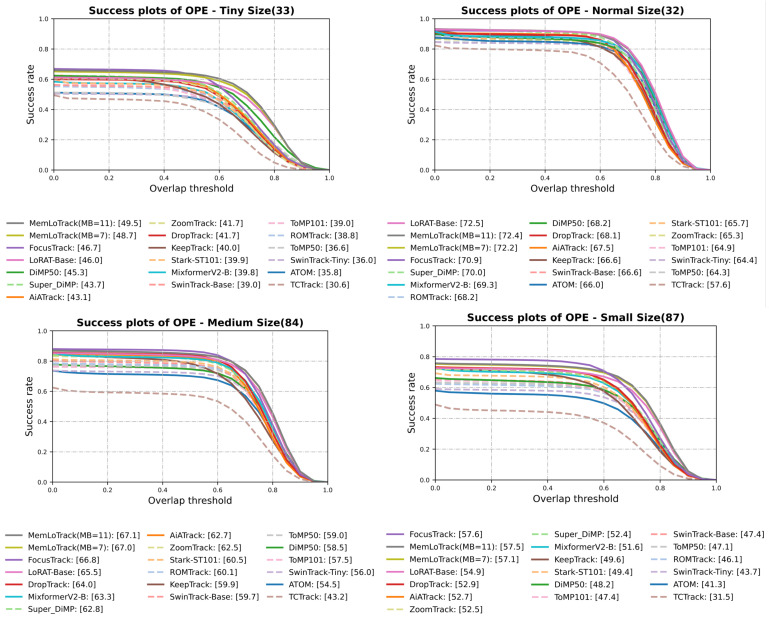
Target-size Success AUC on Anti-UAV410 (Tiny, Small, Medium, Normal).

**Figure 7:**
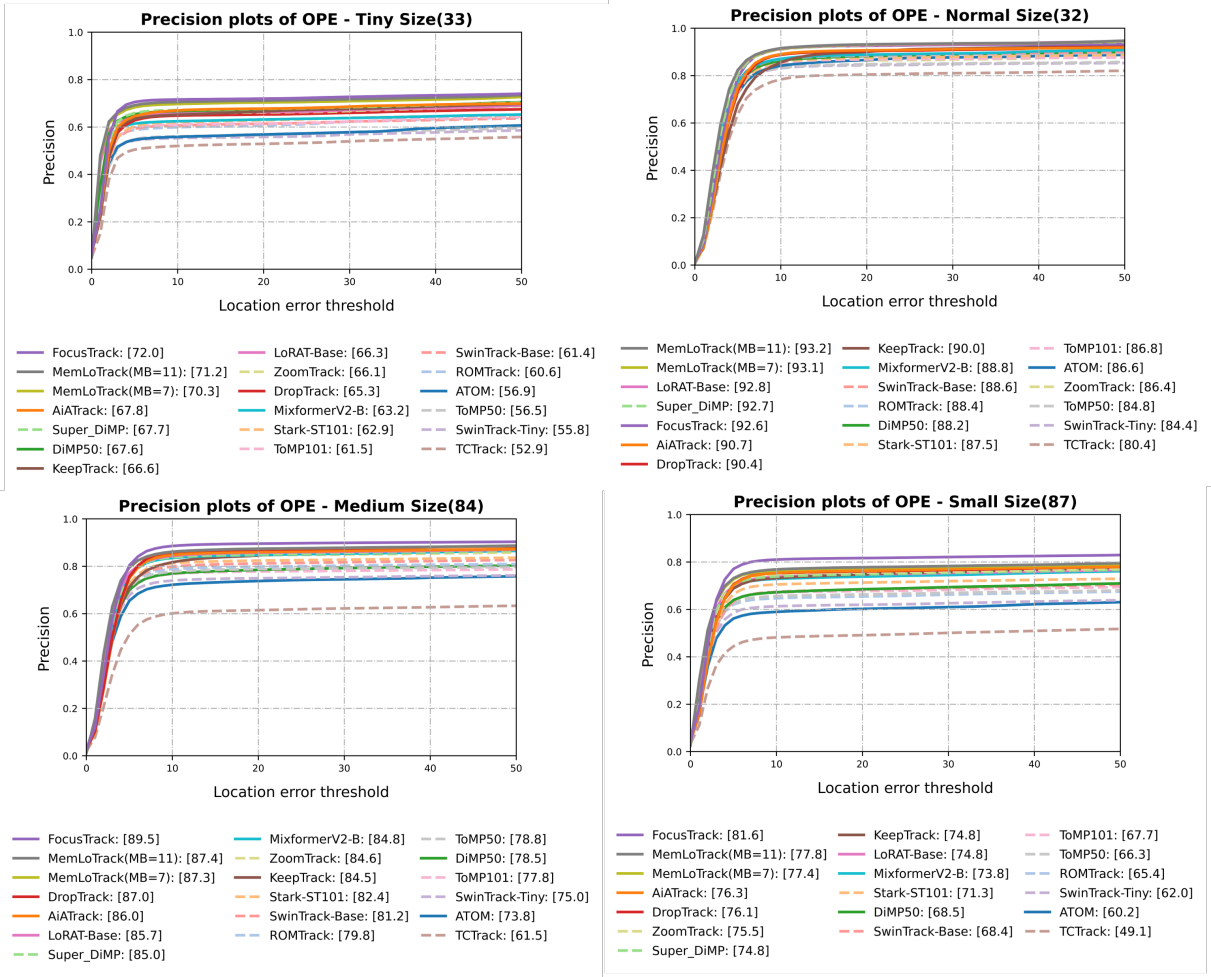
Target-size precision on Anti-UAV410 (Tiny, Small, Medium, Normal).

For a detailed breakdown, Figure 4 and Figure 5 present the attribute-wise Success AUC and precision (at 20 px) comparisons, respectively, for DBC, TC, SV, OC, OV, and FM. Similarly, Figure 6 and Figure 7 summarize the Success AUC and precision results stratified by target size (Tiny, Small, Medium, Normal). As illustrated in Figure 4 and Figure 5, MemLoTrack yields its most significant gains in Success AUC on TC and DBC sequences compared with LoRAT-B and FocusTrack. Figure 5 further reveals that our method slightly trades distance-based precision on FM, OC, and OV for improvements in overall AUC and SA, a behavior consistent with the robustness-oriented design detailed in Section 5. In the size-wise breakdown (Figure 6 and Figure 7), MemLoTrack matches or surpasses FocusTrack across Tiny, Small, Medium, and Normal categories. This highlights that the proposed memory mechanism is particularly beneficial when tracking tiny UAVs with ambiguous thermal signatures.

**Cross-modality evaluation on DUT Anti-UAV RGB tracking dataset.** To assess cross-modality generalization, we evaluated the models on the DUT Anti-UAV RGB dataset [24] using weights pre-trained on general SOT benchmarks, including LaSOT [20], GOT10k [22], TrackingNet [21], and COCO2017 [34]. As shown in Table 3, LoRAT-B [12] outperforms MemLoTrack. This result is expected given the domain shift; unlike thermal imagery characterized by low contrast and Thermal Crossover, RGB targets are texture-rich and visually distinct, enabling robust discrimination based on spatial features alone. In this context, the additional temporal memory offers limited advantage and may introduce unnecessary smoothing. These findings, juxtaposed with the significant gains on the Anti-UAV410 TIR benchmark [2], indicate that our memory-integrated pipeline is inherently specialized for thermal infrared tracking, whereas strong single-frame baselines are more suitable for texture-rich RGB domains.

**Table 2:** Anti-UAV410 test set comparison. In each block, the best per-column score is **bold** and the second best is underlined. Upper block: without training on Anti-UAV410; lower block: after re-training on Anti-UAV410.

Method	Size	Anti-UAV410			SA
		AUC	P	$P_{\mathrm{norm}}$	
*Without training on Anti-UAV410*					
ETTrack [35]	256	41.5	59.7	54.8	41.6
GRM [15]	256	42.3	58.5	55.1	42.2
MixFormerV2-S [7]	224	45.6	64.1	60.0	46.1
ARTrack [16]	256	48.2	67.2	62.9	48.5
TransT [31]	256	48.2	67.7	64.1	48.9
JointNLT [36]	320	48.4	69.0	64.5	48.9
PromptVT [37]	320	50.5	71.5	65.6	51.2
SeqTrack [8]	256	52.2	73.8	70.0	52.9
LoRAT-B [12]	224	**57.2**	77.1	**74**	**57.1**
**MemLoTrack (Ours)**	224	57.1	**77.2**	73.8	56.6
*After re-training on Anti-UAV410*					
TCTrack [38]	287	41.1	60.4	56.0	41.6
SwinTrack-Tiny [9]	224	53.0	71.4	68.1	53.1
OSTrack [6]	256	53.7	73.9	70.9	54.7
ToMP50 [32]	288	54.1	73.8	70.2	55.1
ToMP101 [32]	288	54.2	75.0	70.5	55.1
ROMTrack [39]	256	54.7	74.5	71.7	55.7
SwinTrack-Base [9]	384	55.9	76.4	72.3	55.7
Stark-ST101 [33]	320	56.2	78.5	74.6	57.1
ZoomTrack [40]	256	58.4	81.2	77.4	59.4
AiATrack [41]	320	58.6	82.3	78.0	59.6
MixFormerV2-B [7]	288	58.7	80.5	76.8	59.6
DropTrack [42]	256	59.2	82.2	78.2	60.2
LoRAT-B [12]	224	62.2	80.9	78.1	62.5
FocusTrack [3]	256	62.8	**86.2**	**82.8**	63.9
**MemLoTrack (Ours)**	224	**63.6**	82.7	79.8	**64**

**Table 3:** Comparison with trackers on the DUT Anti-UAV [24] RGB tracking dataset.

Method	Size	AUC	P	$P_{\mathrm{Norm}}$
SiamFC [43]	255	38.1	62.3	52.6
ATOM [30]	288	57.4	83.0	75.8
DiMP [44]	288	57.8	83.1	75.6
TransT [31]	256	58.6	83.2	76.5
LoRAT-B [12]	224	64.1	83.1	87.7
**MemLoTrack (MB = 7)**	224	60.8	78.6	83.4
**MemLoTrack (MB = 11)**	224	63	81.5	86.2

### 4.4. Attribute-Wise Analysis

**Comparison with external trackers.** With MB=7, MemLoTrack demonstrates its strongest performance on the TC and DBC attributes and performs competitively across all target-size buckets (Tiny/Small/Medium/Normal), whereas FocusTrack [3] exhibits superior performance on FM, OC, OV, and SV. This pattern aligns with the aggregate outcome summarized in Section 4.3: MemLoTrack achieves higher AUC and SA but attains lower precision on attributes dominated by abrupt motion, long occlusions, or view losses. In cross-tracker comparisons, FocusTrack’s advantages do not extend to all size buckets; MemLoTrack matches or exceeds it across Tiny/Small/Medium/Normal, while FocusTrack retains its advantage on FM/OC/OV/SV. Specifically, Figure 4 and Figure 5 show that MemLoTrack falls behind on attributes involving rapid displacement (FM) and target loss (OC, OV). This is largely a consequence of the restricted field of view inherent to the 224×224 resolution of the local search window, which hampers the re-acquisition of targets that move out of frame quickly. Furthermore, the linear motion assumption of the Kalman filter is not ideal for abrupt maneuvers, limiting the tracker’s ability to follow erratic UAV flight paths. This limitation regarding kinematic priors is empirically substantiated by our ablation study. Under the FM category, the Kalman-only policy (AUC 48.5) underperforms the visual only Conf-only policy (AUC 48.8), suggesting that kinematic priors can be detrimental when motion is erratic. Moreover, relaxing these constraints (Loose, λ=16.0) yields higher FM performance (AUC 49.7) compared to the Strict setting (λ=4.0, AUC 49.2), confirming that rigid adherence to the Kalman prior hinders tracking effectiveness during sudden acceleration or rotation.

### 4.5. Ablations: Memory and Thresholding

**Effect of MB size.** As established in Section 4.1, our default inference configuration uses MB=7 to align with the 7-frame sampling strategy used during training. Table 4 analyzes the empirical impact of this design choice by comparing it against other bank sizes (MB∈{1,3,7,11}) and the MB=OFF baseline (all at τ=0.8). The baseline (MB=OFF) achieves a strong 62.9 AUC and runs at 424 fps. Enabling memory (MB=1) provides an immediate boost (e.g., +0.6 AUC). Notably, our design-consistent MB = 7 setting also achieves peak performance across AUC (63.6), P (82.7), and P-Norm (79.8), offering the best balance. Increasing the capacity further to MB=11 slightly raises State Accuracy (SA = 64.3), but this configuration exhibits a small drop in AUC and P compared to MB=7. This pattern suggests a mild trade-off. A larger memory bank improves robustness on challenging frames, which is reflected in SA, while providing no additional benefit in per-frame localization accuracy as measured by AUC and P. Moreover, the differences in global AUC and P across MB∈{1,3,7} remain very small, indicating that fine-grained tuning of the memory bank size is not the primary source of the observed gains. Instead, the practical advantage of the memory mechanism becomes clearer when contrasting MB=OFF with MB>0 and examining attribute-wise behavior. As summarized in our attribute-wise results, all MB>0 configurations consistently outperform the memory bank-free baseline under challenging attributes such as OC, OV, and DBC, confirming that the memory bank primarily functions as a robustness mechanism against severe tracking disruptions rather than monotonically improving average AUC and P.

**Attribute-wise trends across MB.** While the overall metrics in Table 4 suggest that MB sizes 1, 3, and 7 yield similar AUC, P, and P-Norm values, the attribute-wise analysis in Table 5 together with the ON/OFF comparison in Table 6 reveals that the memory bank mainly benefits rare but critical failure modes such as occlusion, out-of-view, and Dynamic Background Clutter. In these attributes, switching from MB=OFF to MB=ON yields substantial gains (e.g., OC: +8.2 AUC/+11.4 P), whereas frames from easier segments dominate the dataset-level averages and thus dilute the apparent differences among MB=1, 3, and 7. From a practical standpoint, MB=7 offers the best trade-off between overall accuracy and robustness to such challenging events, while remaining consistent with the training-time memory sampling strategy.

Table 5 details the attribute-level response to MB size (at τ=0.8). As bank size increases from MB=7 to MB=11, performance on challenging attributes improves significantly: **OC** gains +4.3 AUC (26.9 → 31.2) and +5.7 P (50.1 → 55.8). OV, DBC, and FM also see modest gains. This confirms that a larger memory bank enhances robustness to target loss. However, this comes at the cost of performance on SV, which drops by 1.0 AUC (54.1 → 53.1). Performance on Medium and Normal targets peaks at MB=3. This trade-off analysis, combined with the overall metrics in Table 4, solidifies MB=7 as the optimal default.

**Gated memory vs. no memory.** At τ=0.8 and MB=7, enabling the memory bank and Memory Attention Layer yields consistent gains over the no-memory setting (Table 6). The largest deltas (ON−OFF) appear on challenging attributes: OC (+8.2 AUC/+11.4 P), OV (+4.1/+5.9), DBC (+6.5/+9.5), and SV (+3.3/+4.7). Other attributes, such as FM, show marginal changes (−0.1 AUC/−0.1 P), while performance on Medium and Normal size targets remains stable. At the dataset level, enabling the memory bank (MB=ON) improves all metrics (AUC/P/P-Norm/SA) from 62.9/81.8/79.2/63.3 to 63.6/82.7/79.8/64.0.

**Figure 8:**
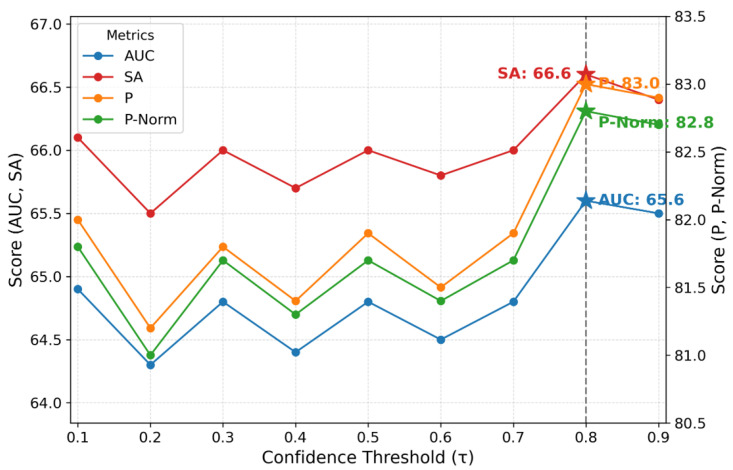
Threshold sweep over τ∈[0.1,0.9] with MB = 7, evaluated on the validation dataset; all metrics peak at τ=0.8.

**Threshold sweep.** We ablate the inference-time confidence gate, sweeping τ∈[0.1,0.9] on the validation split with MB = 7. Tightening τ from 0.8 to 0.9 collapses memory writes from 38,439 to 101 (Table 7), effectively bypassing the Memory Attention Layer and forcing single-frame operation. Despite this severe under-provisioning, the performance at τ=0.9 remains the second-highest (Figure 8), underscoring the tracker’s strong single-frame capabilities. Nevertheless, τ=0.8 provides the optimal balance of filtering and coverage for peak performance across all metrics and is thus adopted as our default.

**Kalman-based motion gating and threshold sensitivity.** To quantify the sensitivity of the proposed motion gate and disentangle the roles of confidence and motion cues, we conducted controlled ablations as summarized in Table 8. The baseline employs an adaptive dual-gating policy with a chi-square threshold of λ=9.21. We compared this against three threshold variants: Strict (λ=4.0), Loose (λ=16.0), and Fixed. Specifically, the Fixed variant is explicitly implemented as a standard non-adaptive Mahalanobis logic; it removes the adaptive scaling mechanism and relies solely on a constant threshold (λ=9.21). We further evaluated two structural ablations: Kalman-only (motion gating without confidence) and Conf-only (confidence gating without motion constraints). All models utilized the same backbone and a fixed memory bank size of MB=7 to ensure fair comparison. The results indicate that the baseline configuration yields the highest State Accuracy (64.0). The threshold variants, including the non-adaptive Fixed model, exhibit only marginal degradation (63.1–63.7), suggesting that the method is robust to hyperparameter fluctuations provided λ is within a reasonable range. In contrast, structural changes lead to significant performance drops. Kalman-only (63.0 SA) underperforms on Tiny targets and SV due to the lack of appearance validation, while Conf-only (62.8 SA) struggles to filter distractors in DBC and OC scenarios without kinematic guidance. These findings confirm that the synergistic combination of confidence and motion priors is critical for robustness in the Anti-UAV410 benchmark.

### 4.6. Qualitative Evaluation

**Figure 9:**
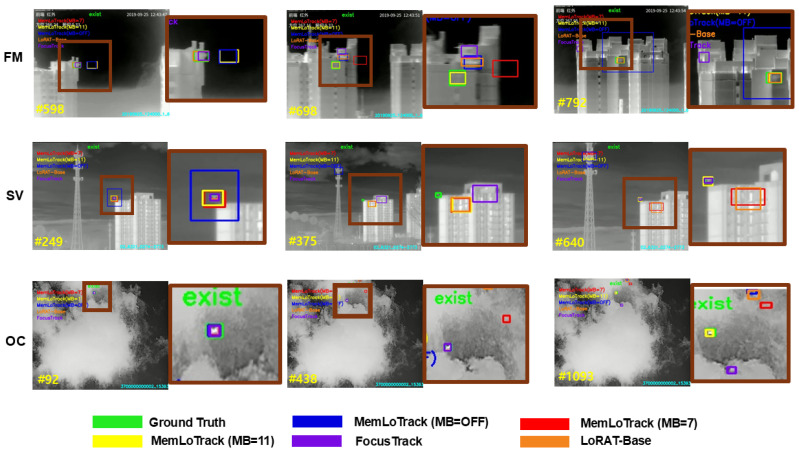
Qualitative comparison on sequences exhibiting Fast Motion (FM), Scale Variation (SV), and occlusion (OC). Outputs from MB = OFF, MB = 7, MB = 11, LoRAT-B, and FocusTrack are shown alongside the ground truth. Rows correspond to one sequence per attribute. Panels alternate between a full frame and a magnified crop; the crop on the right is taken from the region bounded by the brown rectangle in the panel immediately to its left. Inspect at high magnification for fine details.

**Figure 10:**
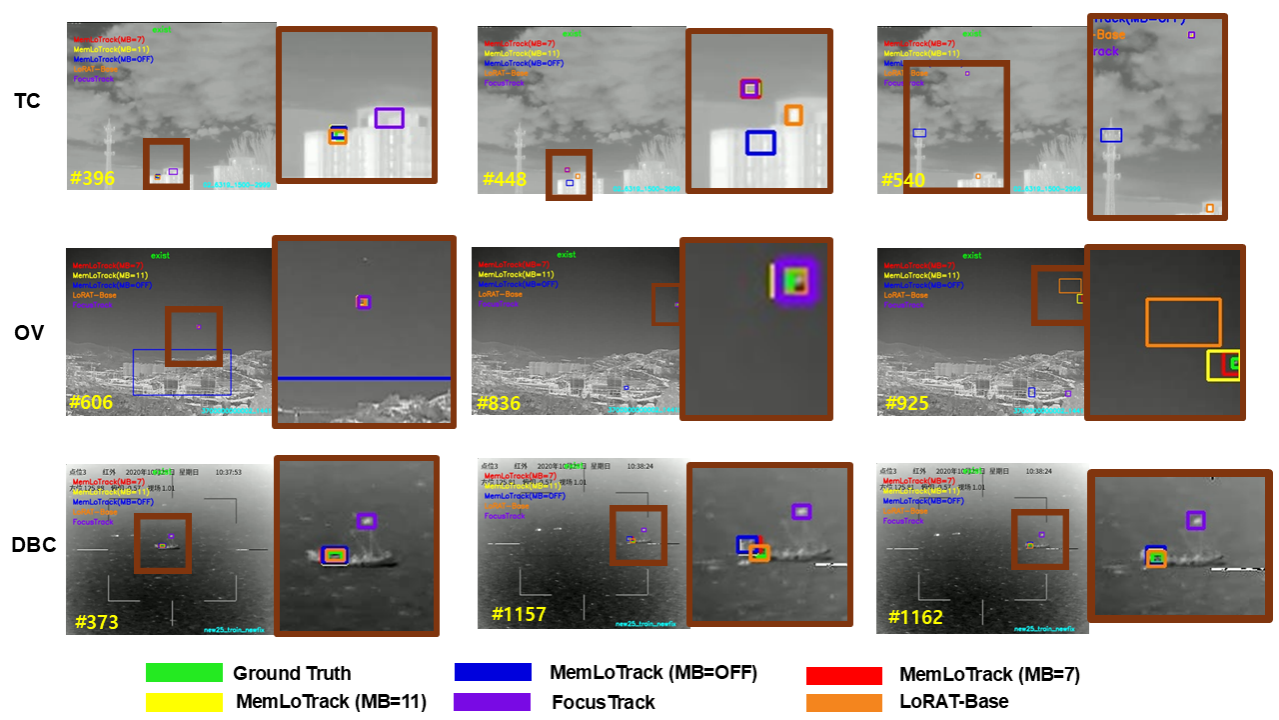
Qualitative comparison on sequences with Thermal Crossover (TC), out-of-view (OV), and Dynamic Background Clutter (DBC). Outputs from MB = OFF, MB = 7, MB = 11, LoRAT-B, and FocusTrack are shown alongside the ground truth. Rows correspond to one sequence per attribute. Panels alternate between a full frame and a magnified crop; the crop on the right is taken from the region bounded by the brown rectangle in the panel immediately to its left. Inspect at high magnification for fine details.

**Visualization setup**. Figure 9 and Figure 10 compare three inference settings of our tracker—MB=OFF,MB=7, and MB=11—together with the LoRAT-B baseline and FocusTrack against the ground truth (GT) across six canonical Anti-UAV410 attributes (FM, SV, OC, TC, OV, DBC). For each attribute, three representative frames are shown with matched zoomed crops to expose fine localization differences. This configuration enables a side-by-side comparison between a single-frame PEFT baseline without explicit temporal memory, a spatially adaptive state-of-the-art tracker, and our memory-augmented variants.

**Effect of memory under occlusion and out-of-view.** When memory is disabled, the tracker is prone to drift during long or partial occlusions and often latches onto distractors after re-entry. A similar drift behavior is observed for the LoRAT-B baseline, which has no explicit temporal memory, whereas FocusTrack generally re-detects onto the target more quickly after occlusion, consistent with its stronger OC and OV scores. Enabling the memory bank markedly stabilizes the trajectory: with MB=7, the tracker reduces post-occlusion jitter and recovers the target with fewer frames; with MB=11, re-acquisition becomes more reliable in prolonged or repeated occlusions. In out-of-view segments, MB=7 suppresses background locks on re-entry, while MB=11 further shortens re-localization lag, reflecting the benefit of longer temporal context. Overall, the qualitative panels show that the memory-enabled variants narrow much of the gap to FocusTrack while clearly improving over LoRAT-B and MB = OFF in OC and OV scenarios.

**Handling of target absence.** In sequences where the target truly leaves the search region (OV), the memory-enabled variants correctly maintain a “no-exist” state until the target reappears, avoiding spurious boxes in clutter. Compared with these variants, LoRAT-B and MB=OFF tend to re-detect the target with a longer delay and exhibit more pronounced localization jitter after re-entry, reflecting the limitations of relying solely on single-frame evidence. FocusTrack and the memory-enabled MemLoTrack variants re-acquire the target more promptly and maintain a more consistent identity once it returns to the field of view. This behavior aligns with the intended presence-aware design of our tracker and is visible in the OV panels: the output remains empty during absence and resumes with a consistent identity upon reappearance.

**Fast Motion and background dynamics.** Under rapid displacement (FM), FocusTrack tends to produce the tightest and most responsive boxes, which reflects its quantitative advantage on this attribute, while the memory bank damps frame-to-frame overshoot, providing steadier box placement than MB=OFF. In the presence of Dynamic Background Clutter (DBC), memory helps discriminate transient background activations from target motion cues; the boxes from MB=7 and MB=11 adhere more tightly to GT than LoRAT-B and FocusTrack, particularly when water, foliage, or heat shimmer introduce confusing patterns.

**Scale Variation and Thermal Crossover.** For steep scale changes (SV), FocusTrack more aggressively follows changes in apparent size and often attains the tightest bounding boxes; MB=7 offers a favorable balance between responsiveness and stability, yielding tighter boxes when altitude or apparent size shifts abruptly. Increasing the bank to MB=11 can retain slightly outdated scale information in rare cases, trading a small amount of responsiveness for robustness to longer context. In Thermal Crossover (TC), both memory-enabled settings diminish target–background ambiguity relative to MB=OFF, LoRAT-B, and FocusTrack, with MB=11 showing marginally cleaner boundaries in sustained crossover intervals.

**Synthesis.** Across attributes, the qualitative evidence supports the same pattern as our quantitative analyses: integrating a memory bank improves robustness to occlusion, out-of-view events, dynamic clutter, and thermal interference while stabilizing localization under Fast Motion. FocusTrack remains the strongest competitor in scenarios dominated by rapid displacement and extended target loss, particularly FM, SV, OC, and OV, whereas MemLoTrack with MB=7 or MB=11 shows clearer advantages in TC and DBC sequences. The LoRAT-B baseline, which relies on single-frame evidence without explicit temporal memory, is especially vulnerable to long occlusions and out-of-view events and frequently exhibits drift or false locks in these cases. Between the two memory budgets, MB=7 provides the best overall balance for precise, scale-responsive localization, whereas MB=11 is preferable when prolonged occlusions or multi-cycle re-entries dominate. In all cases, memory-enabled tracking adheres more closely to GT than MB=OFF, confirming the utility of temporal aggregation in Anti-UAV settings.

### 4.7. Model Size and Computation

We summarize the compute budget of MemLoTrack in Table 9. “Total parameters” counts the entire tracker including the frozen DINOv2 backbone. “Trainable parameters” covers the LoRA adapters, token-type embeddings, the Memory Attention Layer, and the prediction head. “MACs” is the number of multiply-accumulate operations for one forward pass. To further evaluate the efficiency of our approach, we compare MemLoTrack with state-of-the-art trackers in Table 10. While the integration of the memory bank and MAL increases the computational load to 37.5 G MACs compared to the baseline LoRAT-B (30.0 G), MemLoTrack maintains a high inference speed of 153 FPS. Notably, our method is significantly faster than FocusTrack (28 FPS). Although FocusTrack reports lower theoretical MACs, its practical speed is constrained, likely due to the computational overhead of iterative Search Region Adjustment and mask refinement processes. These results demonstrate that MemLoTrack achieves a favorable balance between model complexity and performance, ensuring the real-time capability essential for Anti-UAV applications while delivering robust tracking accuracy.

## 5. Discussion

**Comparison to baseline.** MemLoTrack is developed upon the LoRAT-B-224 architecture and evaluated at an identical input resolution (x=224×224). After fine-tuning on the Anti-UAV410 dataset, our model demonstrates consistent and significant performance improvements over the LoRAT-B baseline across all four standard metrics (e.g., AUC +1.4, P +1.8, P-Norm +1.7, SA +1.5; see Table 2).

**Comparative analysis with state-of-the-art.** Relative to FocusTrack [3], MemLoTrack exhibits a distinct trade-off between robustness and precision on the Anti-UAV410 benchmark. While it trails in distance-based precision (P: 82.7 vs. 86.2), MemLoTrack achieves superior success-oriented metrics, recording higher AUC and SA scores of 63.6 and 64.0 compared to FocusTrack’s 62.8 and 63.9. This divergence reflects our design objective, which prioritizes tracking robustness over pixel-level proximity. We attribute these performance characteristics to the fundamental difference in architectural priors. FocusTrack leverages a spatial prior through Search Region Adjustment and Attention-to-Mask modules to refine localization. Conversely, MemLoTrack implements a temporal prior via a dual-gated FIFO memory and Kalman filtering. By strictly utilizing kinematically consistent frames to guide the Memory Attention Layer, our method excels in attributes such as DBC and TC and remains robust against Tiny targets. In contrast, the spatial adaptability of FocusTrack yields advantages in scenarios with rapid displacement, such as FM, OC, OV, and SV. Although the difference in input resolution (224 vs. 256) may contribute to the precision gap, the results ultimately highlight MemLoTrack as a robustness-oriented alternative that complements the spatial strategies of FocusTrack.

**Effectiveness of the memory mechanism.** The temporal memory is a key contributor to MemLoTrack’s gains under challenging cases. With MB = 7 and τ=0.8, switching memory ON yields the largest OC delta versus MB = OFF (AUC +8.2; P +11.4), with OV and DBC also improving and FM essentially unchanged (Table 6). SA increases modestly, indicating that memory chiefly strengthens presence estimation and target reacquisition.

Varying the memory capacity reveals a trade-off between the benefits of long-term context (recency) and the risk of using outdated information (staleness). Dataset-level AUC and P peak at MB = 7 (Table 4), whereas within MemLoTrack, MB = 11 attains the best absolute OC (AUC 31.2 vs. 26.9 at MB = 7) and slightly higher OV, but lowers overall AUC/P and coincides with a small regression on SV (AUC 54.1→53.1; P 75.0→73.3) (Table 5). On the validation set, a threshold sweep selects τ=0.8 as optimal; τ=0.9 is too strict and degrades performance by delaying memory updates (Figure 8). For completeness, P-Norm is tied at MB = 1 and MB = 7 (79.8), while SA is highest at MB = 11 (64.3).

**Practical implications and guidance.** We use MB = 7 by design—mirroring the seven memory frames used during training—and set τ=0.8 via a validation sweep (Table 4). Prefer MB = 11 only when prolonged occlusion/re-entry dominates and a small loss in fine localization is tolerable. For high-speed maneuvering, memory alone offers limited benefit; compensate with higher input resolution and/or a lightweight motion prior. Throughput remains real-time in our setup (RTX 4070 Ti SUPER); for reference, 153 fps at MB = 7.

## 6. Conclusions

In this work, we presented MemLoTrack, a novel tracking architecture designed for long-horizon robustness. The architecture employs a frozen DINOv2 ViT backbone with parameter-efficient LoRA adapters and a lightweight Memory Attention Layer (MAL) to incorporate temporal context. To align the model with this memory-augmented process, a stateless memory frame sampling strategy is used during training. In contrast, during inference, the temporal context is supplied by a FIFO memory bank, which is critically governed by a dual-gated update policy using both a confidence threshold and a Kalman filter-based motion consistency check. This design effectively curates a high-quality memory to mitigate drift during occlusion and target absence, all without the computational expense of online backbone updates.

On the challenging Anti-UAV410 benchmark, MemLoTrack demonstrates highly competitive performance, achieving the highest reported scores for the key success metrics of AUC (63.6) and SA (64.0). This result represents a significant improvement over the LoRAT-B architecture baseline (+1.4 AUC) and showcases a distinct advantage in overall tracking success when compared to spatially adaptive methods like FocusTrack. This robustness-focused profile, achieved at the cost of some localization precision, validates the effectiveness of our temporally grounded approach for mitigating tracking failures caused by target disappearance and heavy background clutter.

Our comprehensive ablation studies validate that these performance gains are directly attributable to the gated memory mechanism. The temporal context provides pronounced benefits in failure-prone scenarios, most notably under occlusion (+8.2 AUC, +11.4 P) and during out-of-view events, while leaving attributes like Fast Motion largely unaffected. Furthermore, our analysis reveals a clear trade-off between recency and staleness, identifying a memory budget of MB = 7 and a confidence gate of τ=0.8 as the optimal configuration for balancing peak performance with robustness.

Ultimately, MemLoTrack presents a compelling design that balances tracking robustness with computational efficiency. By demonstrating that a selective temporal memory can be integrated into a parameter-efficient framework to achieve real-time throughput (∼153 fps on an NVIDIA RTX 4070 Ti SUPER) without online fine-tuning, our work offers a practical and deployable solution for high-stakes TIR tracking applications. It validates that for complex scenarios dominated by occlusion and clutter, a memory-augmented approach is a powerful strategy, highlighting a promising direction for developing more resilient and efficient visual trackers.

## Figures and Tables

**Table 4:** Overall performance of MemLoTrack with different memory bank (MB) sizes; the best per-column score is **bold**. (fps measured on an NVIDIA GeForce RTX 4070 Ti SUPER.)

Variant	AUC	P	P-Norm	SA	fps
MB = OFF	62.9	81.8	79.2	63.3	424
MB = 1	63.5	82.5	**79.8**	63.5	248
MB = 3	63.4	82.4	79.7	63.7	207
MB = 7	**63.6**	**82.7**	**79.8**	64.0	153
MB = 11	62.5	81.1	78.5	**64.3**	127

**Table 5:** Attribute-wise success (AUC) and precision (P) for MemLoTrack (ours) with different memory bank (MB) sizes.

Attribute	MB = 1		MB = 3		MB = 7		MB = 11	
	AUC	P	AUC	P	AUC	P	AUC	P
Thermal Crossover	59.7	79.7	60.0	80.2	60.5	81.0	60.7	81.1
Out-of-View	46.2	67.6	45.6	66.8	47.1	69.2	48.0	70.0
Scale Variation	53.2	73.4	52.7	72.9	54.1	75.0	53.1	73.3
Fast Motion	49.5	68.3	48.8	67.6	49.5	68.7	50.6	69.8
Occlusion	26.9	50.1	26.9	50.0	26.9	50.1	31.2	55.8
Dynamic Background Clutter	47.9	69.8	47.4	69.1	49.0	71.7	50.0	72.5
Tiny Size	47.8	68.8	46.8	67.5	48.7	70.3	49.5	71.2
Small Size	56.3	76.2	56.7	76.8	57.1	77.4	57.5	77.8
Medium Size	66.7	86.9	67.4	88.0	67.0	87.3	67.1	87.4
Normal Size	72.5	93.3	73.4	94.8	72.2	93.1	72.4	93.2

**Table 6:** Attribute-wise comparison at τ=0.8. MB = ON uses MB size 7; MB = OFF treats the Memory Attention Layer as a no-op. All numbers are percentages. Δ denotes ON−OFF.

Attribute	AUC			P		
	OFF	ON	Δ (ON-OFF)	OFF	ON	Δ (ON-OFF)
Thermal Crossover	59.7	60.5	+0.7	79.7	81.0	+1.3
Out-of-View	43.0	47.1	+4.1	63.3	69.2	+5.9
Scale Variation	50.7	54.1	+3.3	70.3	75.0	+4.7
Fast Motion	49.6	49.5	−0.1	68.8	68.7	−0.1
Occlusion	18.8	26.9	+8.2	38.6	50.1	+11.4
Dynamic Background Clutter	42.5	49.0	+6.5	62.2	71.7	+9.5
Tiny Size	43.5	48.7	+5.1	62.8	70.3	+7.5
Small Size	56.3	57.1	+0.8	76.2	77.4	+1.2
Medium Size	66.9	67.0	+0.1	87.2	87.3	+0.1
Normal Size	72.1	72.2	+0.1	92.8	93.1	+0.3

**Table 7:** Effect of update threshold (τ) on the total number of memory bank updates for MB = 7 across the validation set.

Threshold (τ)	Total Memory Bank Updates
0.1	42,648
0.2	42,105
0.3	41,532
0.4	40,911
0.5	40,250
0.6	39,683
0.7	39,017
0.8	38,439
0.9	101

**Table 8:** Effect of Kalman-based motion gating variants on overall State Accuracy (SA) and attribute-wise AUC/precision on Anti-UAV410. All variants share the same backbone and memory bank size (MB=7, τ=0.8). Only the gating policy differs.

	Ours (dual, λ=9.21)	Strict (λ=4.0)	Loose (λ=16.0)	Fixed (No Adapt.)	Kalman-Only	Conf-Only
State Accuracy (SA)						
SA	64.0	63.1	63.7	63.1	63.0	62.8
Success AUC						
DBC	49.0	47.1	47.4	47.1	47.5	46.0
FM	49.5	49.2	49.7	49.2	48.5	48.8
OC	26.9	26.4	27.0	26.4	26.9	24.3
OV	47.1	45.8	46.3	45.8	44.7	45.0
SV	54.1	53.2	53.6	53.2	50.9	51.5
TC	60.4	59.1	59.6	59.1	59.0	58.7
Tiny	48.7	47.6	47.8	47.6	45.6	46.1
Small	57.1	55.8	56.4	55.8	55.7	55.5
Medium	67.0	66.1	66.7	66.1	66.7	66.4
Normal	72.2	72.4	72.6	72.4	71.7	71.9
Precision (P)						
DBC	71.7	68.7	69.2	68.7	69.4	67.1
FM	68.7	68.0	68.7	68.0	67.1	67.5
OC	50.1	49.5	50.3	49.5	50.3	46.4
OV	69.2	67.0	67.7	67.0	65.6	66.0
SV	75.0	73.6	74.1	73.6	70.1	71.0
TC	81.0	79.1	79.9	79.0	78.9	78.3
Tiny	70.3	68.6	69.0	68.6	65.7	66.5
Small	77.4	75.7	76.5	75.7	75.3	75.1
Medium	87.3	86.2	87.0	86.2	87.0	86.4
Normal	93.1	93.3	93.6	93.3	92.1	92.5

**Table 9:** Model size and computation of MemLoTrack.

Metric	Value
Total parameters (B)	0.12
Trainable parameters (M)	41.34
MACs (G)	37.5

**Table 10:** Comparison with state-of-the-art trackers in terms of computational complexity, inference speed, and tracking accuracy. The inference speeds (FPS) for LoRAT-B and MemLoTrack are measured on an NVIDIA GeForce RTX 4070 Ti Super GPU.

Tracker	Speed (fps)	MACs (G)	AUC	P	SA
OSTrack [6]	267	29.1	53.7	73.9	54.7
ROMTrack [39]	9	34.5	54.7	74.5	55.7
ZoomTrack [40]	276	29.1	58.4	81.2	59.4
DropTrack [42]	154	48.4	59.2	82.2	60.2
FocusTrack [3]	28	30.1	62.8	86.2	63.9
LoRAT-B [12]	700	30.0	62.2	80.9	62.5
**MemLoTrack (MB = 7)**	153	37.5	63.6	82.7	64.0

## Data Availability

The original contributions presented in this study are included in the article. Further inquiries can be directed to the corresponding author.

## References

[1] Jiang N., Wang K., Peng X., Yu X., Wang Q., Xing J., Li G., Guo G., Zhao J., Han Z. (2021). Anti-UAV: A Large Multi-Modal Benchmark for UAV Tracking. arXiv.

[2] Huang B., Li J., Chen J., Wang G., Zhao J., Xu T. (2024). Anti-UAV410: A Thermal Infrared Benchmark and Customized Scheme for Tracking Drones in the Wild. IEEE Trans. Pattern Anal. Mach. Intell..

[3] Wang Y., Xu T., Li J. (2025). FocusTrack: A Self-Adaptive Local Sampling Algorithm for Efficient Anti-UAV Tracking. IEEE Trans. Geosci. Remote Sens..

[4] Huang L., Zhao X., Huang K. Globaltrack: A simple and strong baseline for long-term tracking. Proceedings of the AAAI Conference on Artificial Intelligence.

[5] Voigtlaender P., Luiten J., Torr P.H.S., Leibe B. Siam R-CNN: Visual Tracking by Re-Detection. Proceedings of the 2020 IEEE/CVF Conference on Computer Vision and Pattern Recognition (CVPR).

[6] Ye B., Chang H., Ma B., Shan S. Joint Feature Learning and Relation Modeling for Tracking: A One-Stream Framework. Proceedings of the European Conference on Computer Vision.

[7] Cui Y., Song T.S., Wu G., Wang L. (2023). MixFormerV2: Efficient Fully Transformer Tracking. arXiv.

[8] Chen X., Peng H., Wang D., Lu H., Hu H. Seqtrack: Sequence to sequence learning for visual object tracking. Proceedings of the IEEE/CVF Conference on Computer Vision and Pattern Recognition.

[9] Lin L., Fan H., Xu Y., Ling H. (2021). SwinTrack: A Simple and Strong Baseline for Transformer Tracking. arXiv.

[10] Hu E.J., Shen Y., Wallis P., Allen-Zhu Z., Li Y., Wang S., Wang L., Chen W. (2022). Lora: Low-rank adaptation of large language models. ICLR.

[11] Vaswani A., Shazeer N., Parmar N., Uszkoreit J., Jones L., Gomez A.N., Kaiser Ł., Polosukhin I. (2017). Attention is all you need. Adv. Neural Inf. Process. Syst..

[12] Lin L., Fan H., Zhang Z., Wang Y., Xu Y., Ling H. Tracking Meets LoRA: Faster Training, Larger Model, Stronger Performance. Proceedings of the European Conference on Computer Vision.

[13] Yang C.Y., Huang H.W., Chai W., Jiang Z., Hwang J.N. (2024). SAMURAI: Adapting Segment Anything Model for Zero-Shot Visual Tracking with Motion-Aware Memory. arXiv.

[14] Oquab M., Darcet T., Moutakanni T., Vo H., Szafraniec M., Khalidov V., Fernandez P., Haziza D., Massa F., El-Nouby A. (2023). Dinov2: Learning robust visual features without supervision. arXiv.

[15] Gao S., Zhou C., Zhang J. Generalized Relation Modeling for Transformer Tracking. Proceedings of the 2023 IEEE/CVF Conference on Computer Vision and Pattern Recognition (CVPR).

[16] Bai Y., Zhao Z., Gong Y., Wei X. (2023). ARTrackV2: Prompting Autoregressive Tracker Where to Look and How to Describe. arXiv.

[17] Jia M., Tang L., Chen B.C., Cardie C., Belongie S., Hariharan B., Lim S.N. (2022). Visual prompt tuning. Computer Vision—ECCV 2022, Proceedings of the European Conference on Computer Vision, Tel Aviv, Israel, 23–27 October 2022.

[18] Houlsby N., Giurgiu A., Jastrzebski S., Morrone B., De Laroussilhe Q., Gesmundo A., Attariyan M., Gelly S. Parameter-efficient transfer learning for NLP. Proceedings of the International Conference on Machine Learning.

[19] Liu H., Tam D., Muqeeth M., Mohta J., Huang T., Bansal M., Raffel C.A. (2022). Few-shot parameter-efficient fine-tuning is better and cheaper than in-context learning. Adv. Neural Inf. Process. Syst..

[20] Fan H., Bai H., Lin L., Yang F., Chu P., Deng G., Yu S., Harshit, Huang M., Liu J. (2021). Lasot: A high-quality large-scale single object tracking benchmark. Int. J. Comput. Vis..

[21] Muller M., Bibi A., Giancola S., Alsubaihi S., Ghanem B. Trackingnet: A large-scale dataset and benchmark for object tracking in the wild. Proceedings of the European Conference on Computer Vision (ECCV).

[22] Huang L., Zhao X., Huang K. (2019). Got-10k: A large high-diversity benchmark for generic object tracking in the wild. IEEE Trans. Pattern Anal. Mach. Intell..

[23] Wang X., Shu X., Zhang Z., Jiang B., Wang Y., Tian Y., Wu F. Towards more flexible and accurate object tracking with natural language: Algorithms and benchmark. Proceedings of the IEEE/CVF Conference on Computer Vision and Pattern Recognition.

[24] Zhao J., Zhang J., Li D., Wang D. (2022). Vision-based anti-uav detection and tracking. IEEE Trans. Intell. Transp. Syst..

[25] Liu Q., He Z., Li X., Zheng Y. (2019). PTB-TIR: A thermal infrared pedestrian tracking benchmark. IEEE Trans. Multimed..

[26] Liu Q., Li X., He Z., Li C., Li J., Zhou Z., Yuan D., Li J., Yang K., Fan N. LSOTB-TIR: A Large-Scale High-Diversity Thermal Infrared Object Tracking Benchmark. Proceedings of the 28th ACM International Conference on Multimedia.

[27] Liao D., Shu X., Li Z., Liu Q., Yuan D., Chang X., He Z. (2025). Fine-Grained Feature and Template Reconstruction for TIR Object Tracking. IEEE Trans. Circuits Syst. Video Technol..

[28] Ravi N., Gabeur V., Hu Y.T., Hu R., Ryali C.K., Ma T., Khedr H., Rädle R., Rolland C., Gustafson L. (2024). SAM 2: Segment Anything in Images and Videos. arXiv.

[29] Zheng Y., Zhong B., Liang Q., Mo Z., Zhang S., Li X. Odtrack: Online dense temporal token learning for visual tracking. Proceedings of the AAAI Conference on Artificial Intelligence.

[30] Danelljan M., Bhat G., Khan F.S., Felsberg M. Atom: Accurate tracking by overlap maximization. Proceedings of the IEEE/CVF Conference on Computer Vision and Pattern Recognition.

[31] Chen X., Yan B., Zhu J., Wang D., Yang X., Lu H. Transformer Tracking. Proceedings of the 2021 IEEE/CVF Conference on Computer Vision and Pattern Recognition (CVPR).

[32] Mayer C., Danelljan M., Bhat G., Paul M., Paudel D.P., Yu F., Van Gool L. Transforming Model Prediction for Tracking. Proceedings of the 2022 IEEE/CVF Conference on Computer Vision and Pattern Recognition (CVPR).

[33] Yan B., Peng H., Fu J., Wang D., Lu H. Learning Spatio-Temporal Transformer for Visual Tracking. Proceedings of the 2021 IEEE/CVF International Conference on Computer Vision (ICCV).

[34] Lin T.Y., Maire M., Belongie S., Hays J., Perona P., Ramanan D., Dollár P., Zitnick C.L. (2014). Microsoft coco: Common objects in context. Proceedings of the European Conference on Computer Vision.

[35] Han X., Oishi N., Tian Y., Ucurum E., Young R., Chatwin C., Birch P. (2024). ETTrack: Enhanced temporal motion predictor for multi-object tracking. arXiv.

[36] Zhou L., Zhou Z., Mao K., He Z. Joint Visual Grounding and Tracking with Natural Language Specification. Proceedings of the 2023 IEEE/CVF Conference on Computer Vision and Pattern Recognition (CVPR).

[37] Zhang M., Zhang Q., Song W., Huang D., He Q. (2024). PromptVT: Prompting for Efficient and Accurate Visual Tracking. IEEE Trans. Circuits Syst. Video Technol..

[38] Cao Z., Huang Z., Pan L., Zhang S., Liu Z., Fu C. TCTrack: Temporal Contexts for Aerial Tracking. Proceedings of the 2022 IEEE/CVF Conference on Computer Vision and Pattern Recognition (CVPR).

[39] Cai Y., Liu J., Tang J., Wu G. Robust Object Modeling for Visual Tracking. Proceedings of the 2023 IEEE/CVF International Conference on Computer Vision (ICCV).

[40] Kou Y., Gao J., Li B., Wang G., Hu W., Wang Y., Li L. (2023). ZoomTrack: Target-aware Non-uniform Resizing for Efficient Visual Tracking. arXiv.

[41] Gao S., Zhou C., Ma C., Wang X., Yuan J. (2022). AiATrack: Attention in Attention for Transformer Visual Tracking. arXiv.

[42] Wu Q., Yang T., Liu Z., Lin W., Wu B., Chan A.B. (2023). DropMAE: Learning Representations via Masked Autoencoders with Spatial-Attention Dropout for Temporal Matching Tasks. arXiv.

[43] Bertinetto L., Valmadre J., Henriques J.F., Vedaldi A., Torr P.H. (2016). Fully-convolutional siamese networks for object tracking. Proceedings of the European Conference on Computer Vision.

[44] Bhat G., Danelljan M., Gool L.V., Timofte R. Learning discriminative model prediction for tracking. Proceedings of the IEEE/CVF International Conference on Computer Vision.

